# Decoding HSP90AA1-driven inflammatory signaling in the uveal melanoma microenvironment: an integrated analysis at single-cell resolution

**DOI:** 10.3389/fimmu.2025.1730622

**Published:** 2026-01-06

**Authors:** Yiya Wang, Ziqiu Hu, Min Zhou, Peng Wang

**Affiliations:** 1 Department of Ophthalmology, The First Affiliated Hospital of Chongqing Medical University, Chongqing Key Laboratory for the Prevention and Treatment of Major Blinding Eye Diseases, Chongqing, China; 2 Key Laboratory for Biorheological Science and Technology of Ministry of Education, Bioengineering College of Chongqing University, Chongqing, China; 3 Li Huili Hospital, Ningbo Medical Center, Ningbo, China

**Keywords:** HSP90AA1, inflammation, inflammatory signaling, single-cell resolution, tumor microenvironment, uveal melanoma

## Abstract

**Purpose:**

Decoding key regulators in the uveal melanoma (UVM) tumor microenvironment (TME) is crucial for understanding disease progression and developing novel immunotherapy strategies. This study aims to integrate advanced computational methods and single-cell technologies to identify and validate key molecular regulators mediating inflammatory and immune signal transduction in UVM, and to explore their potential as therapeutic targets.

**Patients and methods:**

An integrated strategy was employed, first utilizing a network-based computational screening approach to identify core regulatory genes associated with UVM progression. Subsequently, single-cell RNA-sequencing (scRNA-seq) data were analyzed to precisely delineate the expression profile of the identified key gene, HSP90AA1, across different cell populations in the UVM microenvironment at single-cell resolution. Finally, the functional role of HSP90AA1 was rigorously validated through siRNA-mediated knockdown, *in vitro* functional assays, and an *in vivo* xenograft model.

**Results:**

Our computational analysis identified HSP90AA1 as a central hub gene. Single-cell analysis revealed that HSP90AA1 is widely expressed across multiple cell types within the UVM tumor microenvironment, particularly in malignant cells, CD8+ T cells, and macrophages. Functional validation confirmed that knockdown of HSP90AA1 significantly suppressed UVM cell proliferation, migration, invasion, and *in vivo* tumor growth. Mechanistically, silencing HSP90AA1 markedly inhibited key inflammatory signaling pathways (e.g., NF-κB, STAT3), leading to a significant reduction in the expression of pro-inflammatory cytokines including TNF-α, IL-6, IL-8, and CCL2, while promoting apoptosis.

**Conclusion:**

By integrating computational biology screening and single-cell resolution analysis, this study successfully decodes HSP90AA1 as a key regulator of the UVM inflammatory and immune microenvironment. These findings, grounded in single-cell insights and confirmed by rigorous experimental validation, reveal the tumor’s intrinsic “chaperone dependency” and highlight HSP90AA1 as a highly promising therapeutic target. Targeting HSP90AA1 may offer a new strategy for modulating the UVM tumor immune microenvironment and overcoming tumor progression.

## 1 Introduction

Uveal melanoma (UVM) is the most common primary intraocular malignancy in adults, accounting for ~3–5% of all melanomas despite its relatively low overall incidence (1). The majority (~85–90%) of UVMs originate from melanocytes in the choroid (2). While excellent local tumor control can be achieved through radiation therapy or enucleation, the long-term clinical outcomes remain poor. Up to 50% of patients eventually develop distant metastases, predominantly in the liver, a rate that has shown little improvement over recent decades (3). Once metastasis occurs, the prognosis is dismal, with a median survival of only 6–13.4 months (4). Currently, no effective systemic adjuvant therapy has been demonstrated to prevent metastasis or significantly improve survival in patients with metastatic UVM (5).

This disconnect between local control and systemic failure underscores UVM’s nature as a systemic disease with ocular manifestations. The management of advanced UVM often involves complex clinical factors, including the administration of potent analgesics like hydromorphone for severe pain management, which is essential for maintaining quality of life (6). Interestingly, the biological actions of such agents—mediated largely by G protein–coupled receptors (GPCRs)—overlap with canonical oncogenic mechanisms in UVM, such as the MAPK/ERK and PI3K/Akt pathways driven by GNAQ and GNA11 mutations (7–11). This signaling convergence suggests that analyzing the intersection between clinical pharmacological targets and tumor pathology could serve as a unique, data-driven entry point to uncover hidden regulatory hubs within the UVM signaling network.

However, systematically identifying these key regulators remains a challenge. While the “opioid paradox” regarding the potential influence of analgesics on tumor biology has been discussed (12–16), few studies have leveraged this interaction network to screen for core molecular drivers in UVM. Network pharmacology offers a systems-level analytical approach to dissect these multidimensional interactions and identify “hub genes” that occupy central regulatory positions.

To bridge this knowledge gap, this study employs an integrated strategy combining computational screening with rigorous experimental validation. We utilized a network-based analysis of the hydromorphone-UVM interaction landscape not merely to evaluate the drug itself, but as a screening filter to pinpoint critical molecular chaperones that sustain the tumor’s malignant phenotype. This approach led to the identification of Heat Shock Protein 90 Alpha Family Class A Member 1 (HSP90AA1) as a central node. HSP90AA1 functions as a “master regulator” stabilizing multiple oncogenic client proteins, including EGFR and ERBB2 (17).

In this study, we aim to decode the intrinsic role of HSP90AA1 in the UVM microenvironment. Specifically, we (1): identified potential core regulatory genes using bioinformatics and network pharmacology screening (2); delineated the expression profile of HSP90AA1 at single-cell resolution across the UVM tumor microenvironment; and (3) performed functional validation to clarify the role of HSP90AA1 in regulating inflammatory signaling and the malignant phenotype. Our findings reveal that HSP90AA1 is not only a central hub gene but also a key driver of “chaperone dependency” in UVM, regulating inflammatory pathways such as NF-κB and STAT3. Targeting HSP90AA1 may therefore offer a promising therapeutic strategy for modulating the UVM immune microenvironment and overcoming disease progression.

## 2 Materials and methods

### 2.1 Data sources and preprocessing

Tumor sample data for the uveal melanoma (UVM) cohort were obtained from The Cancer Genome Atlas (TCGA) database (https://portal.gdc.cancer.gov/). RNA-seq data from normal ocular tissues, serving as controls, were obtained from the Genotype-Tissue Expression (GTEx) database (https://www.gtexportal.org/). All transcriptomic data were normalized using Fragments Per Kilobase of transcript per Million mapped reads (FPKM) expression values for subsequent analyses.

For single-cell–level analysis, this study also integrated UVM samples from four public Gene Expression Omnibus (GEO) datasets: GSE138433, GSE139829, and GSE160883. These datasets originated from multiple research institutions, including the Functional Genomics Platform in Nice-Sophia Antipolis, France; the Miller School of Medicine at the University of Miami; Weill Cornell Medicine. Downstream analyses in this study utilized the processed expression matrices provided by these datasets. In their original studies, single-cell suspensions were prepared immediately after surgical resection of tumor tissues, typically using enzymatic digestion (collagenase and dispase) followed by filtration through strainers of varying pore sizes. Library preparation employed standard commercial kits such as the 10x Genomics 3′ v2 chemistry kit, 5′ VDJ kit, and modified inDrops protocol. Raw sequencing data were aligned and quantified by the original research teams using standard workflows such as Cell Ranger, generating the raw UMI count matrices used in this study. For cell type annotation, we employed a strict dual-validation strategy. First, we aligned our clusters with the high-quality annotations provided by the original authors of the datasets (GSE138433, GSE139829, GSE160883). Second, we verified the identity of malignant cells based on the high expression of canonical melanoma markers (e.g., *MITF, DCT, TYR, MLANA*) and the absence of immune (*PTPRC*) or stromal (*PECAM1*) markers. This approach ensures the accurate identification of malignant populations even in the absence of inferred CNV analysis.

### 2.2 Protein–protein interaction network construction and core gene selection

The target protein list was uploaded to the STRING database (https://string-db.org/) using official gene symbols to construct a protein–protein interaction (PPI) network. The species filter was set to “Homo sapiens,” and the minimum required interaction score was set to 0.4 to ensure interactions with at least moderate confidence. The resulting network data were imported into Cytoscape (version 3.8.2) for visualization and topological analysis.

To identify key nodes within the network, the cytoHubba plugin in Cytoscape was employed to rank all nodes using the Maximal Clique Centrality (MCC) algorithm, which effectively identifies highly connected submodules within the network. The top 10 genes ranked by MCC score were defined as core target genes (hub genes) for subsequent analyses (18).

### 2.3 Functional enrichment analysis

To investigate the potential biological functions of the core target genes, Gene Ontology (GO) and Kyoto Encyclopedia of Genes and Genomes (KEGG) pathway enrichment analyses were performed using the Bioinformatics online platform (https://www.bioinformatics.com.cn/). The GO analysis covered three domains: Biological Process (BP), Cellular Component (CC), and Molecular Function (MF). The top 10 enriched terms with the smallest *p*-values were visualized. KEGG analysis was used to identify the main signaling pathways associated with the core target genes.

### 2.4 Expression validation and prognostic value analysis of core targets

To validate the expression patterns of the core targets in UVM and assess their clinical prognostic value, differential expression and survival analyses were conducted using R software (version 4.3.2). Expression differences of HSP90AA1 between TCGA-UVM tumor samples and GTEx normal ocular tissues were evaluated using the Wilcoxon signed-rank test.

Prognostic analyses were based on clinical follow-up data from the TCGA-UVM cohort. First, univariate Cox regression was applied to assess the association between each core target gene and patient prognosis, calculating hazard ratios (HR) and 95% confidence intervals (CIs). Second, Kaplan–Meier survival curves were generated, and log-rank tests were performed to compare overall survival (OS), disease-specific survival (DSS), and progression-free interval (PFI) between high- and low-expression groups, stratified by the median expression value.

### 2.5 Single-sample gene set enrichment analysis

To evaluate the functional activity and prognostic significance of the core target gene set as a functional unit, single-sample gene set enrichment analysis (ssGSEA) was performed using the GSVA R package. An ssGSEA enrichment score was calculated for each TCGA-UVM sample, representing the comprehensive expression activity of the core target gene set. Patients were then divided into high- and low-score groups according to the median enrichment score, followed by Kaplan–Meier survival analysis.

### 2.6 Multidimensional bioinformatics analysis of HSP90AA1

#### 2.6.1 Alternative splicing analysis

Alternative splicing (AS) events of HSP90AA1 across the TCGA pan-cancer cohort were analyzed using the TCGA SpliceSeq database (https://bioinformatics.mdanderson.org/TCGASpliceSeq). Splicing patterns across various cancer types were investigated by comparing the Percent Spliced In (PSI) values of distinct splicing events.

#### 2.6.2 Tumor immune microenvironment association analysis

To explore the relationship between HSP90AA1 and the tumor immune microenvironment (TIME), several analyses were conducted. First, the TISIDB platform (http://cis.hku.hk/TISIDB/) was used to examine correlations between HSP90AA1 expression and multiple immune-related molecules, including immune stimulatory and inhibitory genes, chemokines, and HLA genes.

Second, based on the pan-cancer immune subtyping system proposed by Thorsson et al., we analyzed the distribution of HSP90AA1 expression across immune subtypes (C1–C6) and further evaluated its associations with immune-related features such as leukocyte infiltration, IFN-γ response, and TCR diversity.

#### 2.6.3 Cancer immune cycle and immunotherapy potential analysis

The TIP database (http://biocc.hrbmu.edu.cn/TIP/) was employed to assess Spearman correlations between HSP90AA1 expression and the activity of the seven steps of the cancer immune cycle. Additionally, the easier R package was used to compute multiple biomarker scores predicting immune checkpoint blockade (ICB) efficacy (e.g., CYT, TLS, IFN-γ signature, etc.), and differences in these scores between high- and low-HSP90AA1 expression groups were compared.

### 2.7 Human protein atlas database validation

To further validate HSP90AA1 expression and localization at both the transcriptional and protein levels, data from the Human Protein Atlas (HPA) database (version 21.0, https://www.proteinatlas.org/) were analyzed. Specifically, we examined:

1. Transcriptional expression (nTPM) of HSP90AA1 across normal tissues, immune cells, and cancer cell lines;
2. Spearman correlations between HSP90AA1 expression and immune cell infiltration;
3. Subcellular localization of HSP90AA1 protein by analyzing downloaded immunofluorescence (IF) images.

### 2.8 Single-cell transcriptome data analysis

To delineate HSP90AA1 expression patterns at single-cell resolution, multiple UVM single-cell transcriptomic datasets from **GEO** (https://www.ncbi.nlm.nih.gov/geo/) were downloaded and integrated. Data were normalized, dimensionally reduced (PCA), and clustered using the Seurat R package. Cells were visualized with Uniform Manifold Approximation and Projection (UMAP).

To mitigate expression sparsity resulting from “dropout” events in single-cell data, the Nebulosa R package was applied to compute weighted kernel density estimations, thereby smoothing expression signals and providing a more accurate representation of HSP90AA1 expression distribution in the UMAP space (19).

### 2.9 Cell culture and treatment

Human uveal melanoma cell lines C918 and MUM-2B were obtained from The Cell Bank of Chinese Academy of Sciences (Beijing, China) and cultured in RPMI-1640 medium (Gibco, USA) supplemented with 10% fetal bovine serum (FBS) at 37 °C in a humidified atmosphere containing 5% CO_2_. Cells were routinely passaged every 2–3 days and used for subsequent experiments in the logarithmic growth phase.

### 2.10 Real-time quantitative polymerase chain reaction

Total RNA was extracted from C918 and MUM-2B cells using TRIzol reagent (Invitrogen, USA) according to the manufacturer’s protocol. Complementary DNA (cDNA) was synthesized using the RevertAid First Strand cDNA Synthesis Kit (Thermo Fisher Scientific, USA). Real-time PCR amplification was performed using SYBR Green PCR Master Mix (Thermo Fisher Scientific, USA) on the ABI StepOnePlus™ Real-Time PCR System (Applied Biosystems, USA).The cycling conditions were as follows:

95°C for 5 min, followed by 40 cycles of 95°C for 15 sec and 60°C for 20 sec.

Gene expression was normalized to GAPDH, and relative expression levels were calculated using the 2^-^ΔΔCt method. The primers used in this study were as follows:

HSP90AA1-F: 5′-TATAAGGCAGGCGCGGGGGT-3′.

HSP90AA1-R: 5′-TGCACCAGCCTGCAAAGCTTCC-3′.

GAPDH-F: 5′-GAAGGTGAAGGTCGGAGTC-3′.

GAPDH-R: 5′-GAAGATGGTGATGGGATTTC-3′.

All reactions were performed in triplicate, and the specificity of amplification was confirmed by melt curve analysis.

### 2.11 Cell viability assay

Cell viability was assessed using the CCK-8 assay (Beyotime, China). Cells were seeded into 96-well plates (5×10³ cells/well) and incubated for 24 h. After treatment, 10 μL of CCK-8 reagent was added per well and incubated for 2 h. Absorbance was measured at 450 nm using a microplate reader (Bio-Rad, USA). Experiments were repeated in six replicates.

### 2.12 Colony formation assay

C918 and MUM-2B cells were transfected with siHSP90AA1-1, siHSP90AA1-2, siHSP90AA1-3, or siNC. After transfection, 500 cells per well were seeded in 6-well plates in 1 mL of complete RPMI-1640 medium and cultured at 37°C in a 5% CO^-^ incubator for 14 days to allow colony formation. At the end of the incubation period, the medium was removed, and the cells were fixed with 4% paraformaldehyde (Sinopharm Group Chemical Reagent Co., Ltd.) for 10 min at room temperature, followed by staining with 0.1% crystal violet solution (Solarbio, Beijing, China) for 15 min. After washing with PBS and drying, colonies were photographed under a light microscope. Colonies containing more than 50 cells were counted, and the average colony number per group was calculated from five randomly selected fields. All experiments were performed in triplicate.

### 2.13 Wound-healing assay

C918 and MUM-2B cells were seeded into 12-well plates at a density of 2 × 10^5^ cells per well in 200 μL of complete RPMI-1640 medium and incubated for 24 h to reach near confluency. A scratch was made across the cell monolayer using a sterile 10 μL pipette tip, followed by gently washing twice with PBS to remove floating cells. Cells were then cultured in serum-free RPMI-1640 medium for another 24 h. Images of the scratched area were captured immediately after wounding (S_24_h) and after 24 h of incubation (S_24_h) using an inverted microscope. The relative wound distance (%) was calculated using the formula:


$$({\text{S}}_{0}\text{h}\;-\;{\text{S}}_{24}{\text{h)/S}}_{0}\text{h}\;\times \;100\%.$$

Each group included cells transfected with siHSP90AA1-1, siHSP90AA1-2, siHSP90AA1-3, and a negative control (NC). All assays were performed in triplicate and quantified using ImageJ software.

### 2.14 Transwell migration and invasion assays

The migration and invasion capacities of C918 and MUM-2B cells were evaluated using Transwell chambers (8 μm pore size; Corning, COSTAR, China).For the invasion assay, the upper chambers were pre-coated with 0.5 mg/mL Matrigel (Corning, 356234) at 4°C for 12 h, then rinsed with 0.5 mL of serum-free RPMI-1640 medium. Approximately 2 × 10^4^ cells in 200 μL of serum-free medium were seeded into the upper chambers, while 500 μL of RPMI-1640 medium containing 10% FBS was added to the lower chambers as a chemoattractant. After 24 h of incubation at 37°C, non-invading cells on the upper surface of the membrane were gently removed with a cotton swab. The invaded cells on the lower surface were fixed with 4% paraformaldehyde (Sinopharm Group, China) for 10 min and stained with 0.1% crystal violet (Solarbio, Beijing, China) for 15 min at room temperature. Stained cells were imaged under a light microscope, and five random fields per insert were counted for quantification. For the migration assay, the protocol was identical to the invasion assay except that the Matrigel coating step was omitted. Each condition included cells transfected with siHSP90AA1-1, siHSP90AA1-2, siHSP90AA1-3, and a negative control (NC). All experiments were independently repeated three times.

### 2.15 Enzyme-linked immunosorbent assay

A human IL-6 ELISA kit (ab178013), human TNF-α ELISA kit (ab181421), human STAT3 ELISA kit (ab176655), NF-κB p65 Transcription Factor Assay Kit (ab133112), human IL-8 ELISA kit (ab214030), human MCP-1/CCL2 ELISA kit (ab179886), human Bcl-2 ELISA kit (ab272102), human Bax ELISA kit (ab199080), and human Caspase-3 ELISA kit (ab285337) were purchased from Abcam (UK). The concentrations of IL-6, TNF-α, STAT3, NF-κB p65, IL-8, MCP-1, Bcl-2, Bax, and Caspase-3 in the conditioned medium of cultured cells were measured in triplicate by ELISA according to the manufacturer’s instructions. Briefly, the supernatant from treated cells was collected, filtered through a 0.22 μm membrane, and incubated with HRP-conjugated detection antibodies in pre-coated 96-well plates. After washing, chromogenic substrate was added for signal development. Absorbance was measured at 450 nm using a microplate reader, and cytokine concentrations were calculated based on the standard curves provided.

### 2.16 *In vivo* tumor xenograft assay

Four-week-old male BALB/c nude mice were purchased from GemPharmatech Co., Ltd. (Jiangsu, China). All animal procedures were performed in accordance with the guidelines of the Institutional Animal Care and Use Committee of Hangzhou Institute of Medicine, Chinese Academy of Sciences.For the tumor formation assay, C918 cells (2 × 10^6^) stably transfected with either shNC or shHSP90AA1-3 lentivirus were suspended in 100 μL of serum-free RPMI-1640 medium and subcutaneously injected into the right flank of nude mice (n = 5 per group). Tumor volumes were measured every 5 days using digital calipers and calculated using the formula:V = ½ × L × W², where L represents the longest diameter and W represents the shortest diameter. After 28 days, all mice were humanely euthanized by CO_2_ inhalation. The tumors were excised, photographed, and weighed.

### 2.17 Statistical analysis

All data were analyzed using SPSS 18.0 (IBM, USA). Results are presented as mean ± standard deviation (SD) from at least three independent experiments. For comparisons between two groups, Student’s t-test was used when data were normally distributed; otherwise, the Mann–Whitney U test was applied. For comparisons among multiple groups, one-way ANOVA followed by SNK *post-hoc* test or Kruskal–Wallis test was used depending on data distribution. Chi-square test or Fisher’s exact test was used for categorical variables. A p-value less than 0.05 was considered statistically significant.

## 3 Results

### 3.1 Identification of potential hub genes in UVM via network pharmacology screening

**Figure 1:**
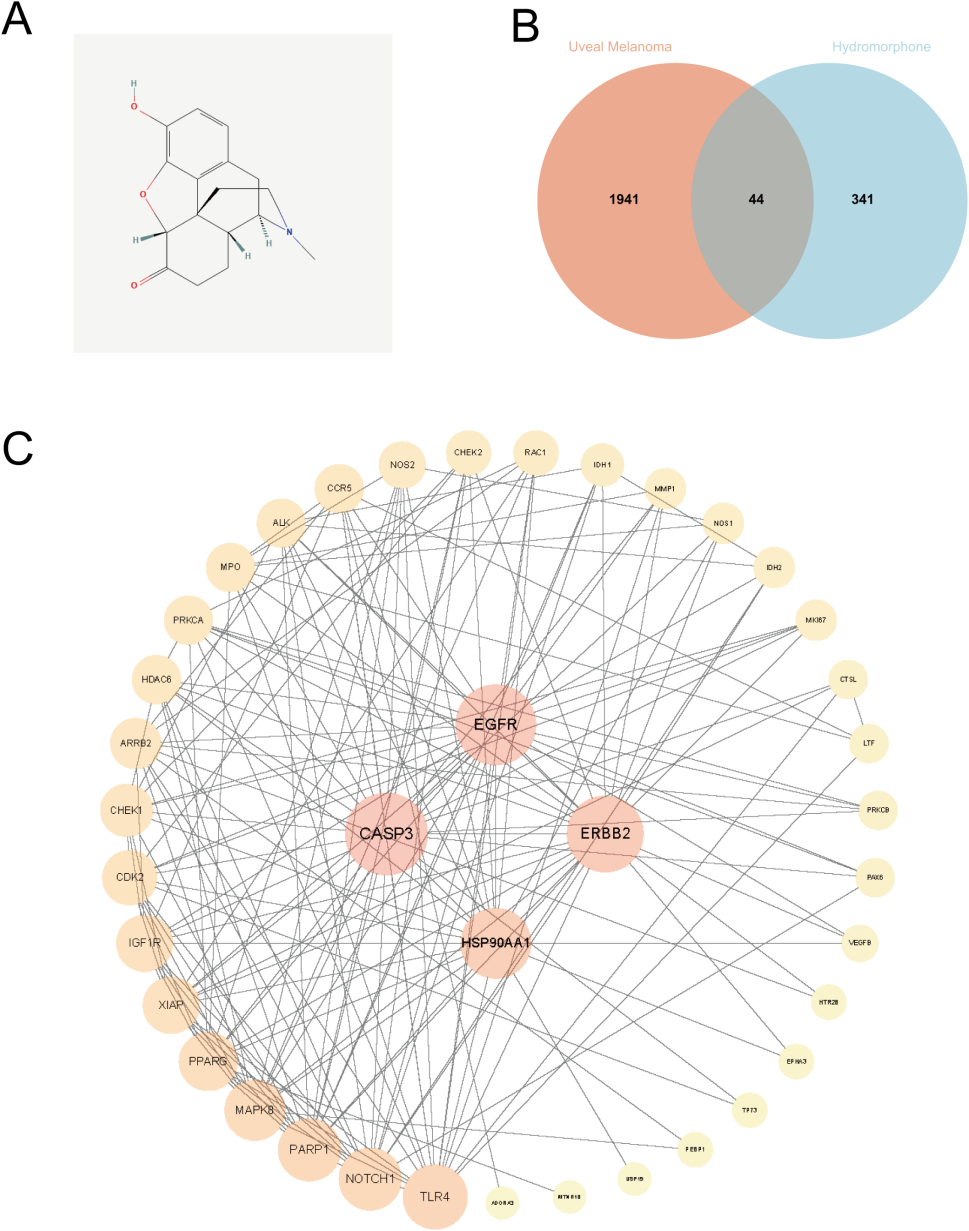
Identification of shared targets and construction of protein-protein interaction network. **(A)** 2D chemical structure of hydromorphone. **(B)** Venn diagram illustrating the 44 common gene targets identified between hydromorphone and uveal melanoma (UVM). **(C)** A protein-protein interaction (PPI) network of the 44 shared targets, constructed using the STRING database. Nodes represent proteins, and edges represent the interactions between them.

To explore the potential pharmacological effects of hydromorphone on uveal melanoma (UVM), target prediction was first performed. By integrating results from multiple databases, a total of 385 potential protein targets for hydromorphone were identified. In parallel, a search of the GeneCards database yielded 1,985 genes associated with UVM development and progression. Intersection analysis of these two datasets identified 44 overlapping targets shared by hydromorphone and UVM (**Figures 1A, B**), providing a foundation for subsequent functional analyses.

### 3.2 PPI network construction and core gene screening

**Figure 2:**
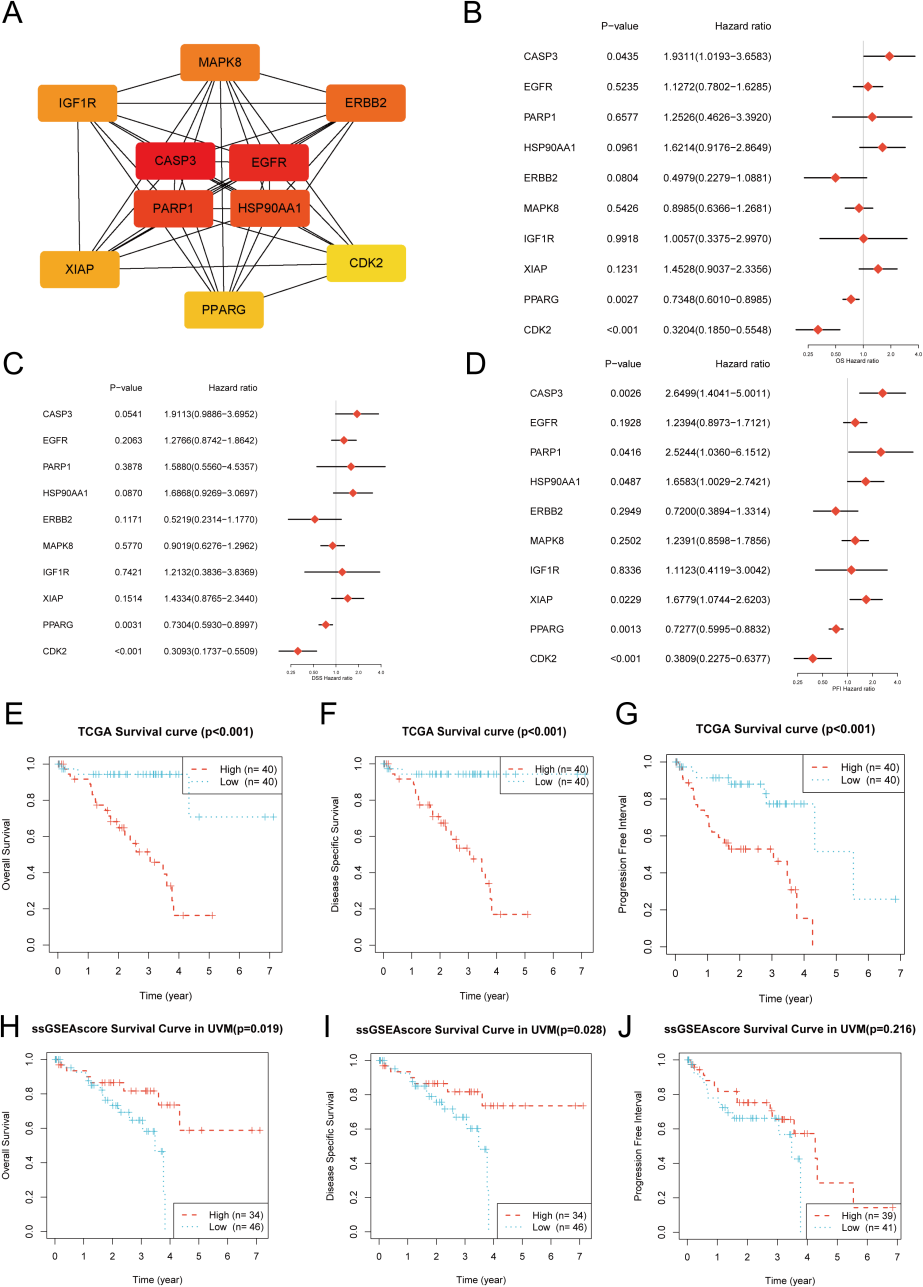
Hub gene identification and clinical prognostic value in uveal melanoma. **(A)** The protein-protein interaction (PPI) subnetwork of the 10 identified hub genes. **(B–D)** Forest plots from univariate Cox regression analysis showing the hazard ratios (HR) and prognostic significance of the 10 hub genes for Overall Survival (OS), Disease-Specific Survival (DSS), and Progression-Free Interval (PFI). **(E–G)** Kaplan-Meier survival curves for OS, DSS, and PFI, comparing patients with high versus low expression of the 10-gene signature, based on data from The Cancer Genome Atlas (TCGA). **(H–J)** Kaplan-Meier survival curves for OS, DSS, and PFI based on single-sample Gene Set Enrichment Analysis (ssGSEA) scores for the hub gene signature.

To systematically elucidate the functional relationships among these 44 shared targets, a protein–protein interaction (PPI) network was constructed (**Figure 1C**). The network revealed complex interconnections among target proteins. To identify central genes occupying key regulatory positions, topological analysis was performed using the cytoHubba plugin in Cytoscape software. Based on the Maximal Clique Centrality (MCC) algorithm, the top 10 genes ranked by MCC scores were identified as core (hub) genes: MAPK8, EGFR, ERBB2, IGF1R, CASP3, HSP90AA1, PARP1, XIAP, PPARG, and CDK2. The subnetwork formed by these hub genes represented a highly interconnected functional module (**Figure 2A**).

### 3.3 Functional enrichment analysis of core target genes

To elucidate the biological processes and signaling pathways involving the 10 core targets, Gene Ontology (GO) and Kyoto Encyclopedia of Genes and Genomes (KEGG) enrichment analyses were performed.

At the biological process (BP) level, the core genes were predominantly enriched in inflammation- and stress-related processes such as peptidylserine phosphorylation, response to lipopolysaccharide, and cellular response to abiotic stimuli.

At the cellular component (CC) level, they were mainly localized in endosomal, secretory granule, and vesicular compartments.

**Figure 3:**
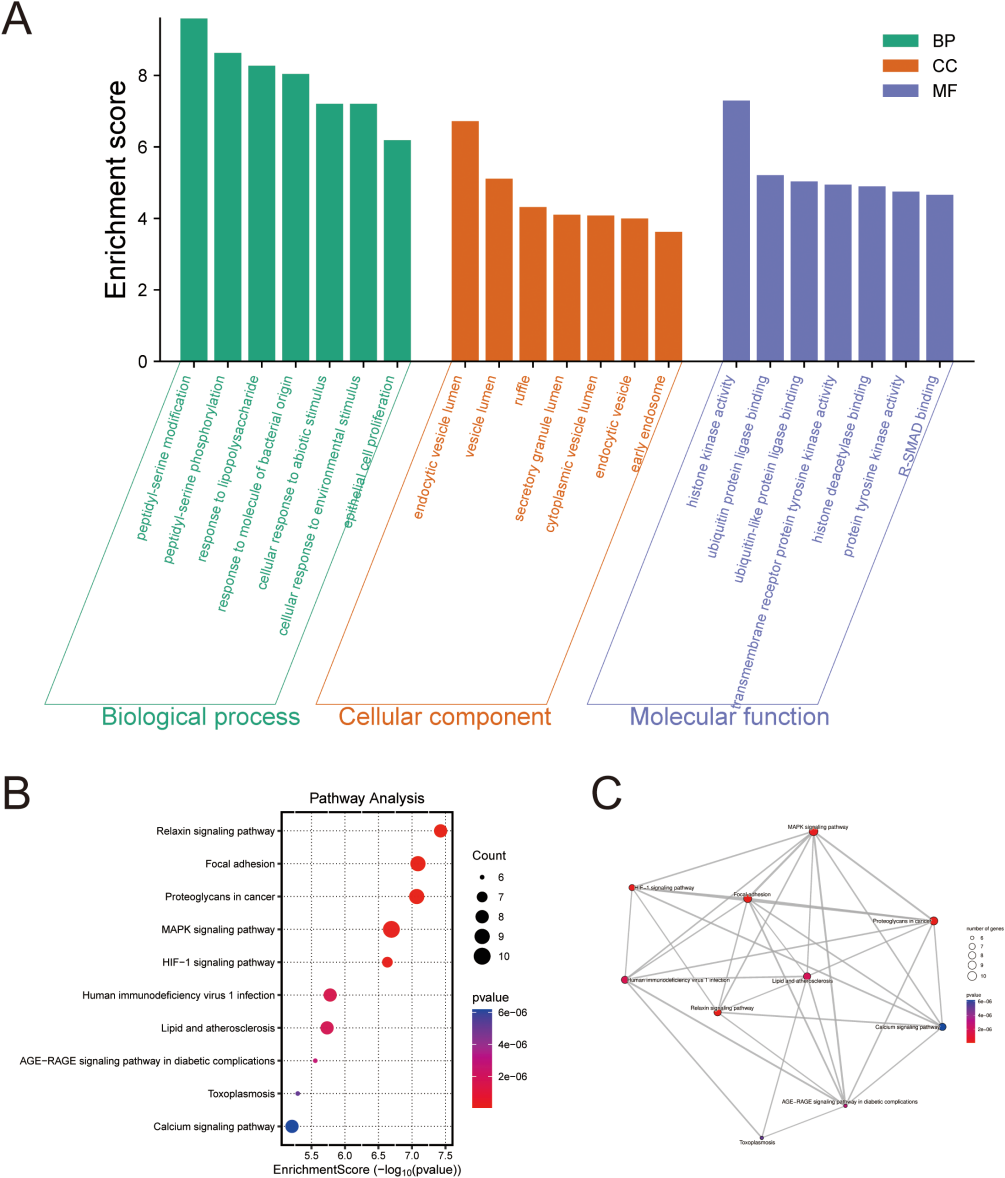
Functional enrichment analysis of hub genes. **(A)** Gene Ontology (GO) analysis of the 10 hub genes, showing the top enriched terms for Biological Process (BP), Cellular Component (CC), and Molecular Function (MF). **(B)** Bubble plot of Kyoto Encyclopedia of Genes and Genomes (KEGG) pathway analysis. The bubble size corresponds to the number of genes enriched in a pathway, and the color indicates the statistical significance (p-value). **(C)** A network plot visualizing the interconnections between the significantly enriched KEGG pathways.

At the molecular function (MF) level, enriched terms included histone kinase activity, ubiquitin-like ligase binding, and receptor tyrosine kinase binding (**Figure 3A**).

KEGG pathway analysis further revealed significant enrichment in tumorigenesis- and progression-related pathways, including proteoglycan synthesis, MAPK signaling, HIF-1 signaling, and central carbon metabolism in cancer (**Figures 3B, C**).

These findings suggest that hydromorphone may modulate key biological processes—such as inflammation, cell proliferation, and metabolism—by targeting these hub genes in UVM.

### 3.4 Clinical prognostic value of core targets

To determine the clinical relevance of the core targets, their prognostic value was evaluated using the TCGA-UVM cohort.

Univariate Cox regression analysis revealed that among the 10 hub genes, only CASP3 expression was significantly correlated with progression-free interval (PFI), with higher expression indicating a favorable prognosis (HR = 0.38, 95% CI [0.17–0.85], p < 0.05). Expression levels of the remaining genes showed no statistically significant association with overall survival (OS), disease-specific survival (DSS), or PFI (**Figures 2B–D**).

However, when the 10 hub genes were analyzed collectively as a gene set, a strong prognostic association emerged. Kaplan–Meier survival analysis demonstrated that patients with high expression of the core gene set had significantly shorter OS, DSS, and PFI compared with the low-expression group (p < 0.001) (**Figures 2E–G**). Similarly, single-sample gene set enrichment analysis (ssGSEA) revealed that higher ssGSEA scores—representing greater activity of the core gene module—were significantly associated with poorer survival outcomes. Kaplan–Meier curves indicated reduced OS (p = 0.019) and DSS (p = 0.028) in the high-score group (**Figures 2H, I**), suggesting that this hydromorphone-related functional module may act synergistically to promote UVM malignancy.

### 3.5 Expression pattern and clinical significance of the core gene HSP90AA1

**Figure 4:**
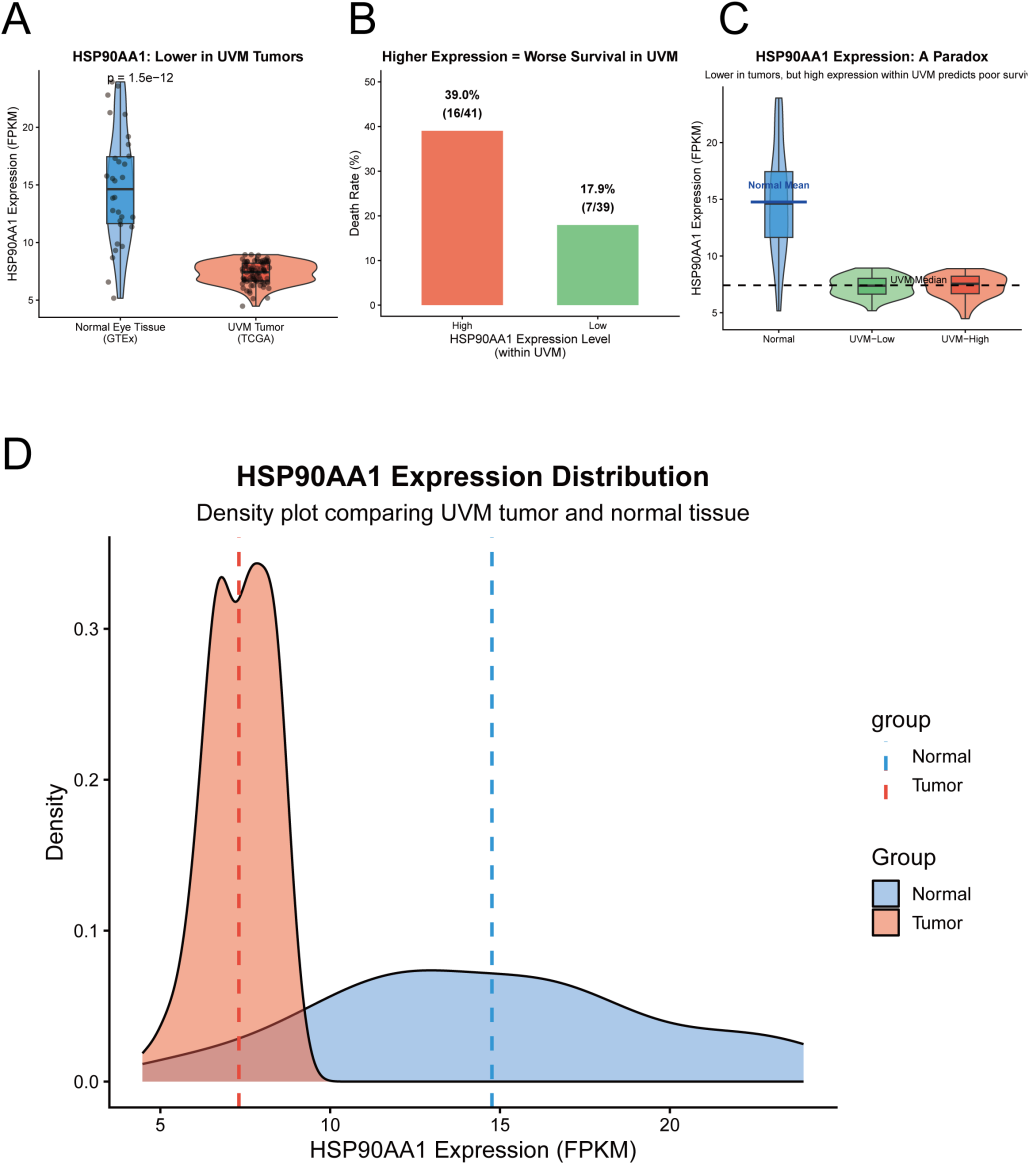
HSP90AA1 expression patterns and prognostic paradox in uveal melanoma. **(A)** Violin plot comparing HSP90AA1 transcript levels in UVM tumor samples (TCGA) and normal ocular tissues (GTEx), showing significant downregulation in tumors (p = 1.5e-12). **(B)** Bar chart illustrating a higher death rate in UVM patients with high intratumoral HSP90AA1 expression compared to the low-expression group. **(C)** A graphical representation of the HSP90AA1 paradox, where its expression is lower in tumors than in normal tissue, yet higher expression within the tumor cohort predicts worse survival. **(D)** Density plot comparing the expression distribution of HSP90AA1 between normal (blue) and tumor (red) tissues.

Given its fundamental role in protein homeostasis and cellular stress regulation, HSP90AA1 was selected for in-depth functional analysis. Comparative analysis of TCGA and GTEx datasets revealed significantly decreased transcriptional levels of HSP90AA1 in UVM tumor tissues compared with normal ocular tissues (p = 1.5 × 10^-12^) (**Figures 4A-D**).

Interestingly, HSP90AA1 exhibited a prognostic paradox within the UVM cohort: although its overall expression was reduced in tumors, patients with higher HSP90AA1 expression (39.0%) displayed markedly higher mortality rates than those with lower expression (17.9%) (**Figures 4B, C**).

### 3.6 Pan-cancer alternative splicing and immune microenvironment association of HSP90AA1

**Figure 5:**
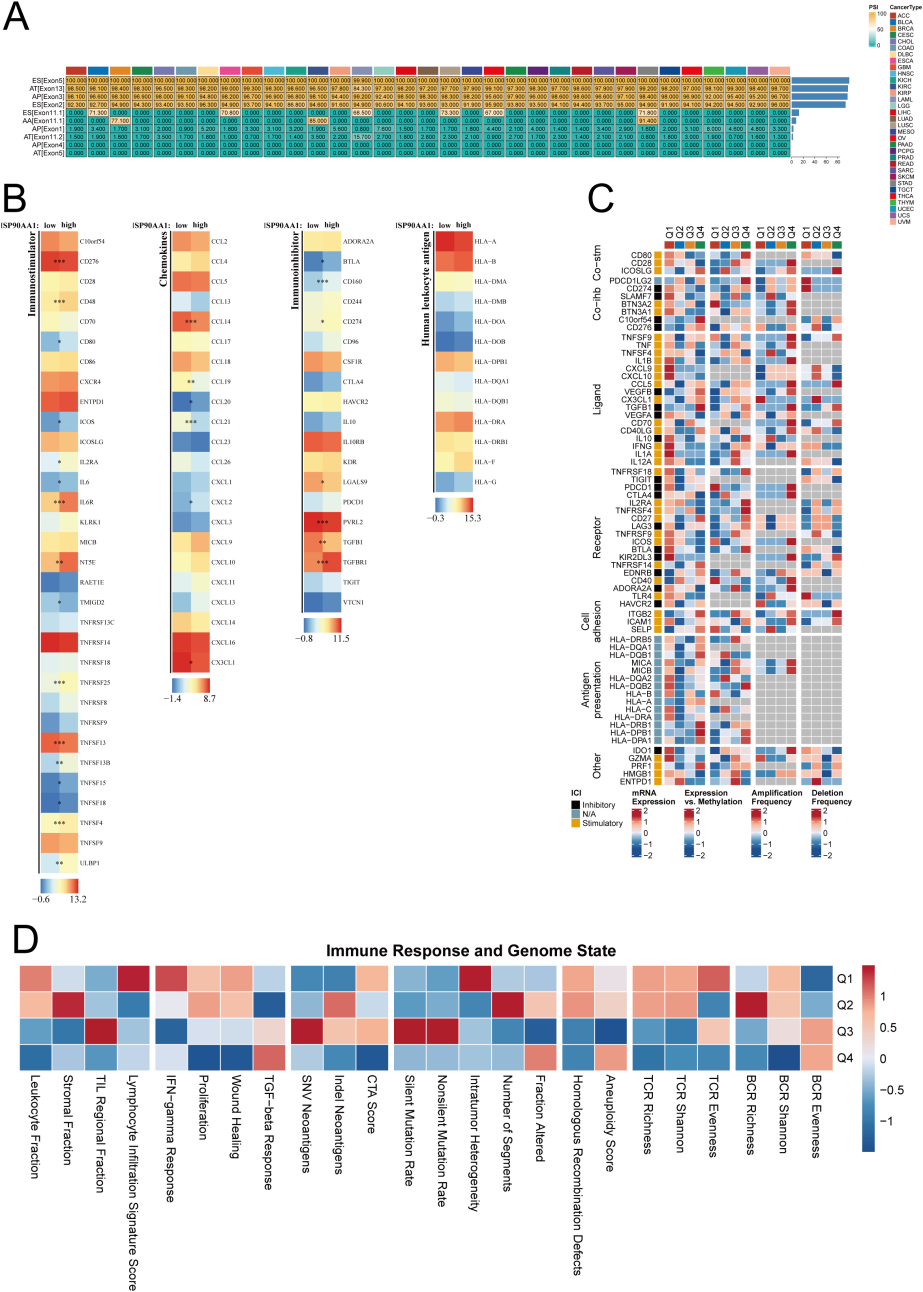
Pan-cancer and immune microenvironment analysis of HSP90AA1. **(A)** An overview of alternative splicing (AS) events for HSP90AA1 across multiple cancer types. **(B)** Heatmaps illustrating the differential expression of immunostimulatory molecules, chemokines, immunoinhibitory molecules, and MHC genes between high- and low-HSP90AA1 expression groups in UVM. **(C)** A detailed heatmap showing expression patterns of various immune-related genes associated with HSP90AA1 levels. **(D)** Heatmap showing the association between HSP90AA1 expression quartiles (Q1-Q4) and the status of key immune response and genomic features in the UVM microenvironment. * p < 0.05, ** p < 0.01, and *** p < 0.001.

To further explore the immunological role of HSP90AA1, a pan-cancer alternative splicing (AS) analysis was performed. Multiple AS events—including exon skipping (ES) and alternative donor sites (AD)—were identified, showing cancer-specific splicing patterns (**Figure 5A**).

In UVM, HSP90AA1 expression was tightly associated with the tumor immune microenvironment (TIME). Heatmap analysis showed that in the high-HSP90AA1 group, several immune stimulatory molecules (e.g., CD86, TNFSF4) and chemokines (e.g., CXCL9, CXCL10) were significantly upregulated, whereas certain immunosuppressive genes (e.g., TGFB1, PDCD1LG2) were downregulated (**Figure 5B**).

High HSP90AA1 expression (Q1 subtype) was also correlated with increased leukocyte infiltration, enhanced IFN-γ response, and elevated neoantigen load (**Figure 5D**).

### 3.7 Pan-tissue expression profile, immune infiltration correlation, and subcellular localization of HSP90AA1

**Figure 6:**
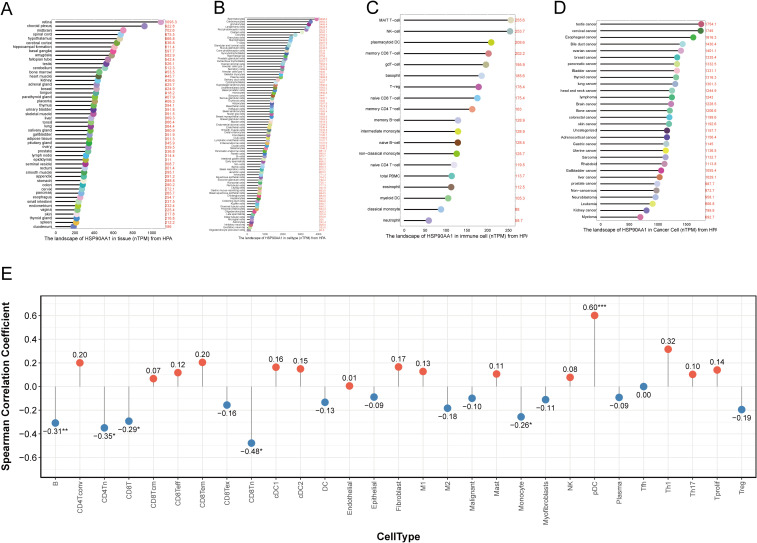
Pan-tissue expression profile and immune infiltration correlation of HSP90AA1. Data is from the Human Protein Atlas (HPA) database. **(A)** mRNA expression of HSP90AA1 across a panel of normal human tissues. **(B)** Expression across various human cell types. **(C)** Expression within different immune cell populations. **(D)** Expression across a range of cancer cell lines. **(E)** A plot of Spearman correlation coefficients between HSP90AA1 expression and the infiltration levels of different immune cell types in the UVM tumor microenvironment.

The Human Protein Atlas (HPA) database was used to comprehensively characterize HSP90AA1 expression across normal and tumor tissues. HSP90AA1 exhibited widespread expression across multiple human systems, with particularly high abundance in metabolically active organs such as the brain, adrenal glands, and testes (**Figure 6A**). Similarly, consistent expression was observed across various cell types and cancer cell lines (**Figures 6B, D**).

Within the immune system, HSP90AA1 expression was notably high in plasmacytoid dendritic cells (pDCs) and natural killer (NK) cells (**Figure 6C**).

Spearman correlation analysis demonstrated a strong positive association between HSP90AA1 expression and pDC infiltration (R = 0.60, p < 0.001), and a significant negative correlation with the infiltration of B cells, CD4^+^ T cells, and CD8^+^ T cells (**Figure 6E**). These findings indicate that HSP90AA1 may participate in shaping the immunosuppressive microenvironment of UVM.

**Figure 7:**
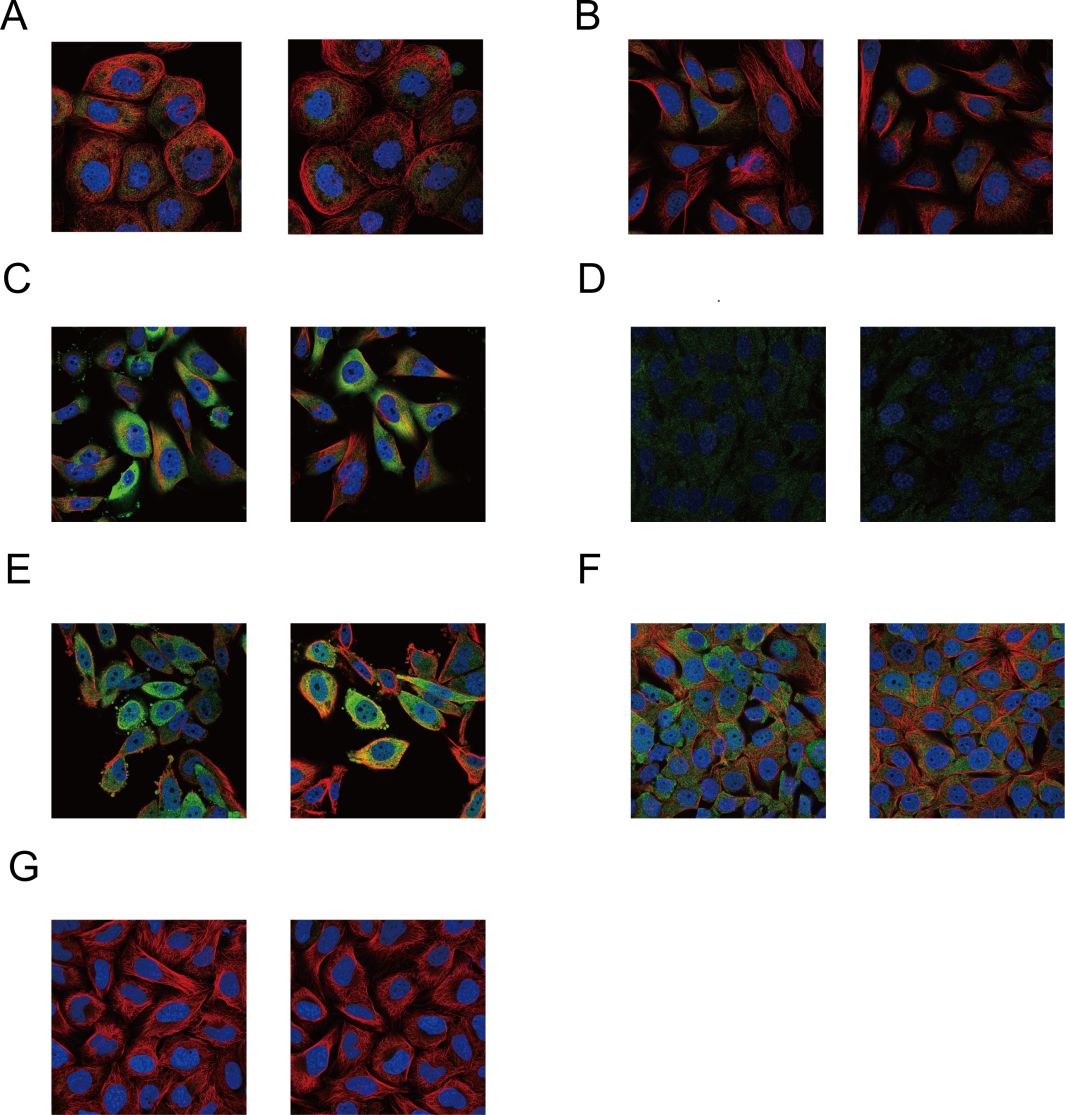
Subcellular localization of HSP90AA1 protein in human cell lines. Representative immunofluorescence images from the Human Protein Atlas. **(A–C)** Staining using antibody CAB002058 in: **(A)** A-431 cells, **(B)** U2OS cells, and **(C)** U-251 MG cells. **(D–G)** Staining using antibody HPA047290 in: **(D)** hTERT-RPE1 cells (serum starved), **(E)** PC-3 cells, **(F)** MCF-7 cells, and **(G)** U2OS cells. The images show HSP90AA1 localization (green), microtubules (red), and nuclei (blue).

At the protein level, immunofluorescence (IF) images from HPA confirmed cytoplasmic localization of HSP90AA1 (green signal), showing distinct co-localization with tubulin (red) (**Figure 7**).

### 3.8 Single-cell expression profiling of HSP90AA1 in the UVM tumor microenvironment

**Figure 8:**
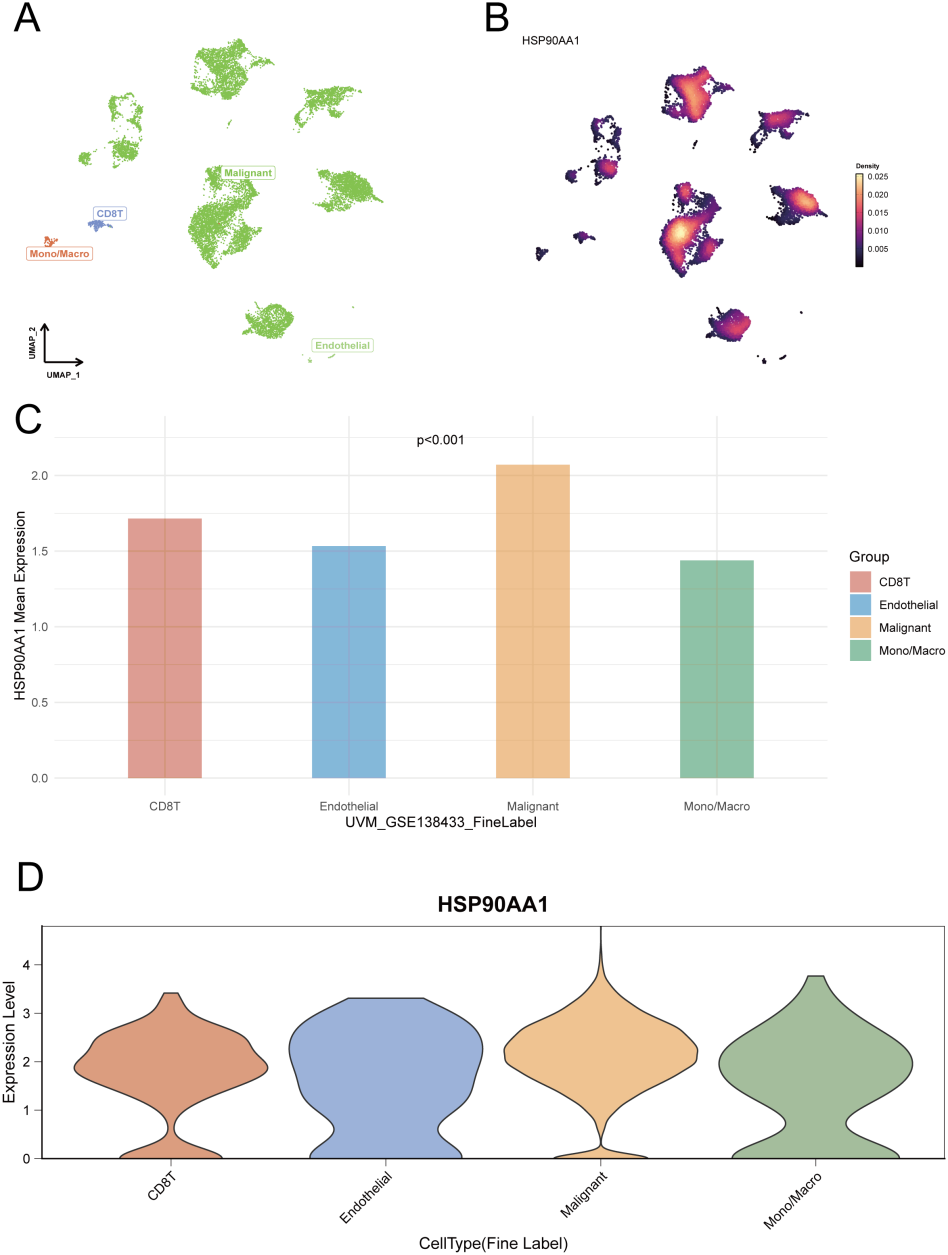
Single-cell expression profiling of HSP90AA1 in the UVM tumor microenvironment (Dataset GSE138433). **(A)** UMAP (Uniform Manifold Approximation and Projection) plot showing identified cell clusters, including malignant, endothelial, CD8+ T (CD8T), and mono/macrophage cells. **(B)** A Nebulosa density plot visualizing the expression level and distribution of HSP90AA1 across the UMAP space. **(C)** Bar graph comparing the mean expression of HSP90AA1 across the main cell types. **(D)** Violin plots depicting the expression distribution of HSP90AA1 within each cell population.

**Figure 9:**
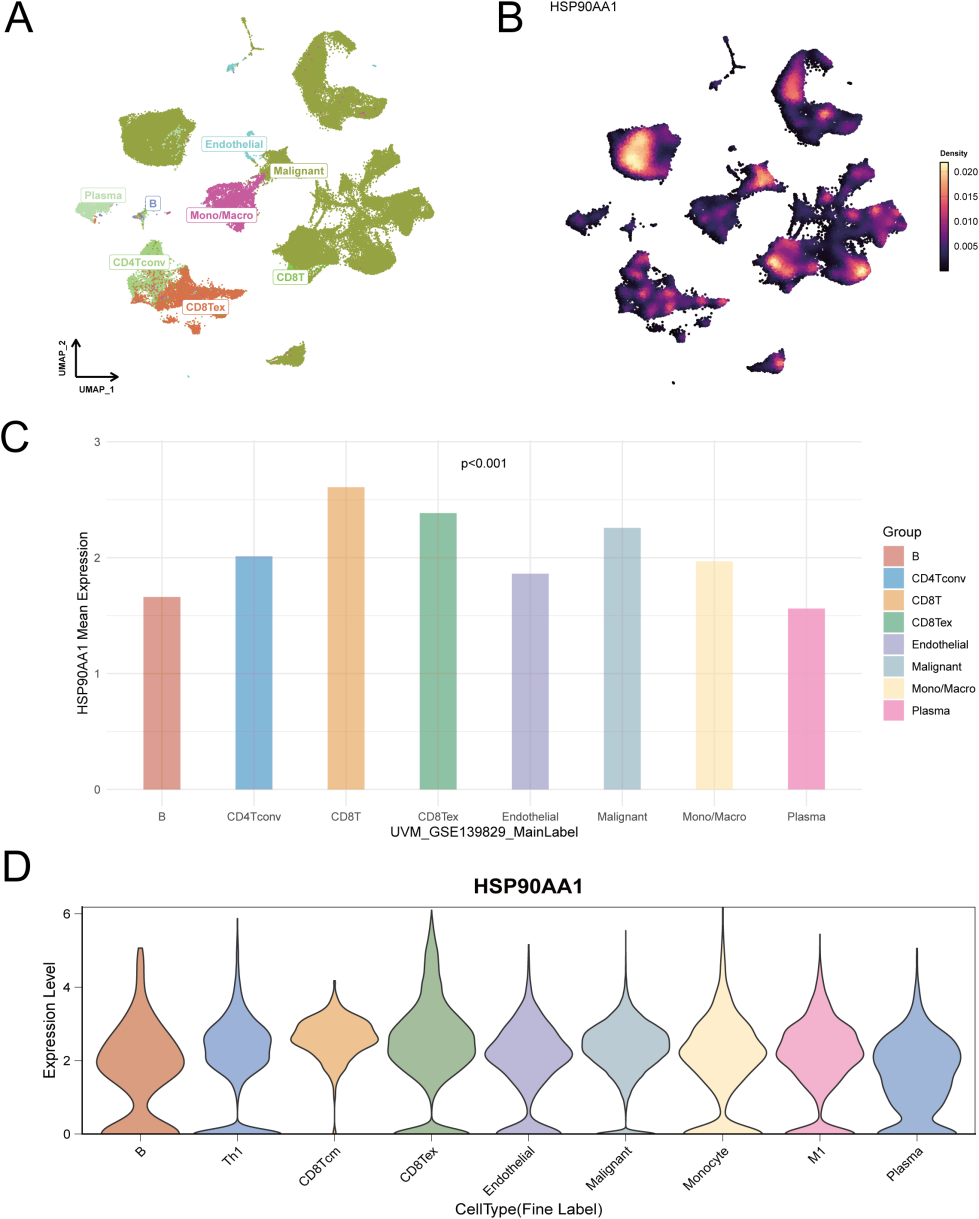
Single-cell resolution of HSP90AA1 expression in a second UVM cohort (Dataset GSE139829). **(A)** UMAP plot identifying distinct cell populations within the tumor microenvironment, including malignant cells and various immune subsets. **(B)** A corresponding Nebulosa feature plot illustrating the density of HSP90AA1 expression across all cells. **(C)** Bar chart quantifying the mean expression of HSP90AA1 across major cell lineages. **(D)** Violin plots showing the expression distribution of HSP90AA1 across more detailed cell subtypes.

**Figure 10:**
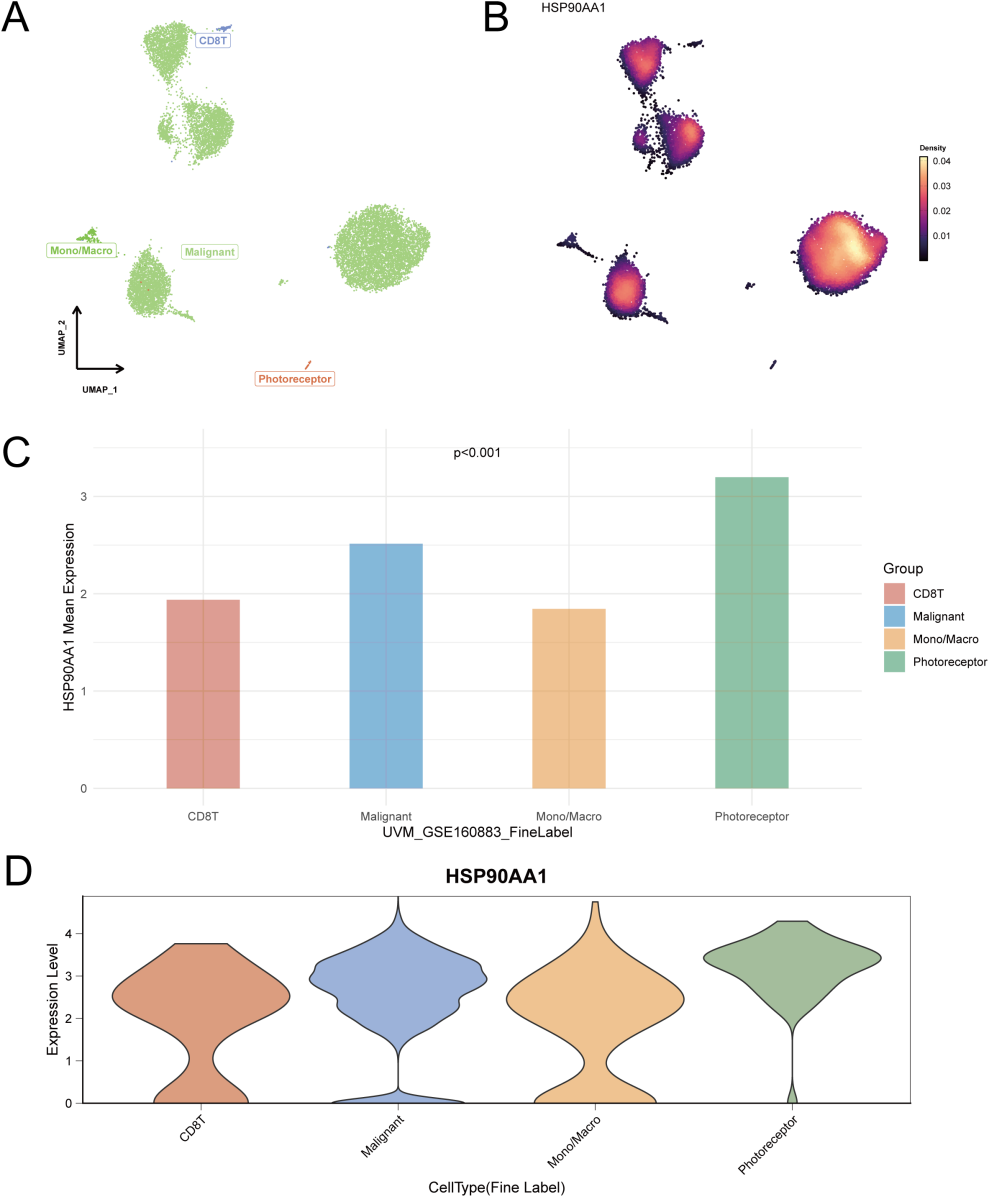
Single-cell expression analysis of HSP90AA1 in a third UVM cohort (Dataset GSE160883). **(A)** UMAP visualization of cell clusters identified in the tumor microenvironment, including malignant cells, photoreceptors, CD8+ T cells (CD8T), and monocytes/macrophages (Mono/Macro). **(B)** A Nebulosa density plot showing the distribution and expression intensity of HSP90AA1 across the identified cell populations. **(C)** Bar graph representing the mean expression of HSP90AA1 in each major cell type. **(D)** Violin plots illustrating the expression distribution of HSP90AA1 within the distinct cell lineages.

To delineate HSP90AA1 expression at single-cell resolution, four independent UVM scRNA-seq datasets were analyzed. UMAP dimensionality reduction successfully identified major cell populations within the tumor microenvironment (TME), including malignant cells, immune cells (CD8^+^ T cells, macrophages/monocytes, B cells), and endothelial cells (**Figures 8**–**10**).

Using Nebulosa density mapping to address data sparsity, we found that HSP90AA1 was expressed across multiple cell types rather than confined to a specific population. Quantitative comparisons revealed higher expression in malignant tumor cells, CD8^+^ T cells, and macrophages/monocytes, indicating a broad cellular expression pattern and suggesting that HSP90AA1 may contribute to UVM progression by modulating diverse cell populations within the TME.

### 3.9 HSP90AA1 knockdown suppresses malignant phenotypes of UVM cells

**Figure 11:**
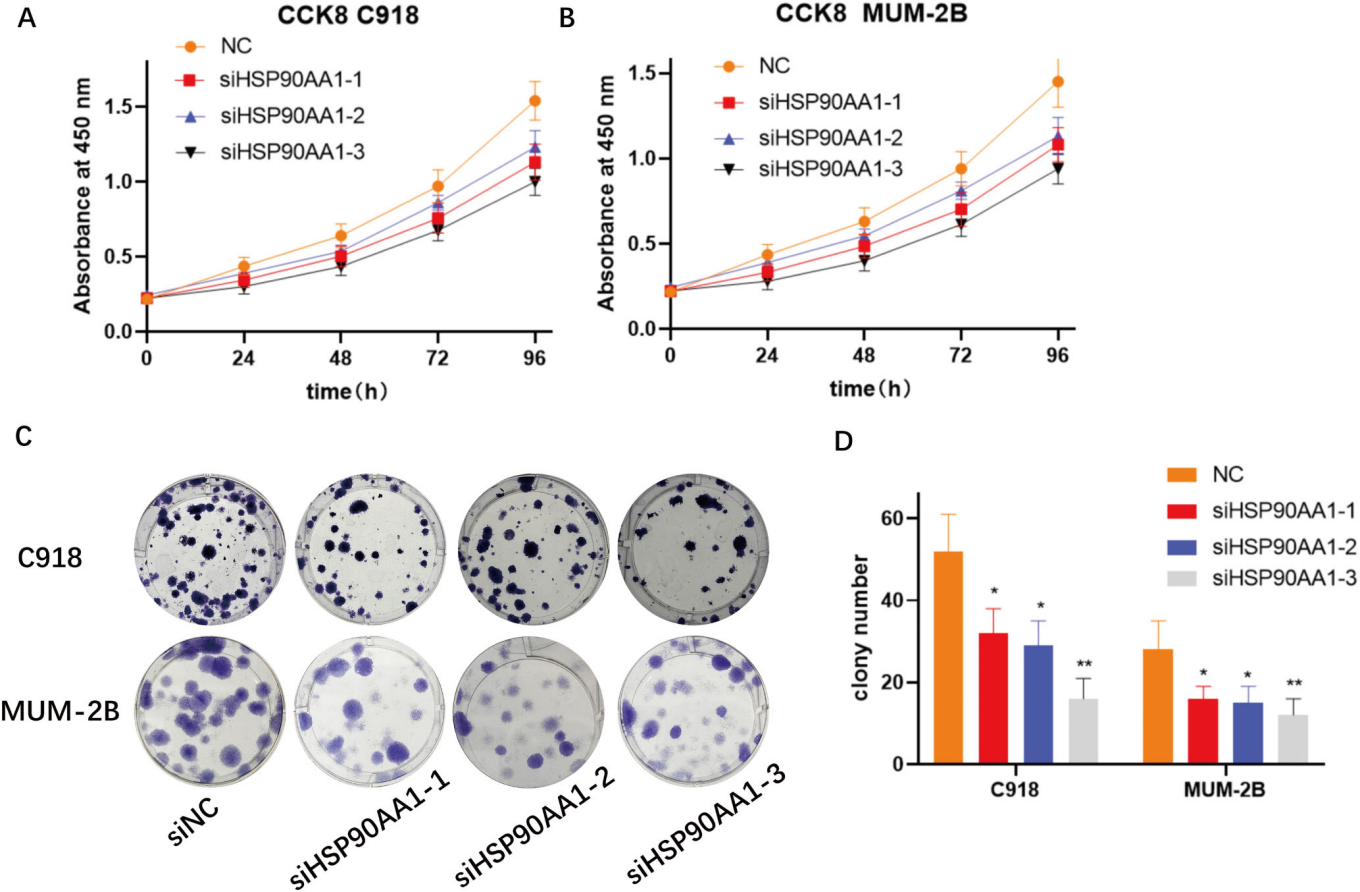
Silencing HSP90AA1 inhibits proliferation and colony formation in UVM cells. **(A, B)** Cell viability of C918 and MUM-2B cells was assessed using the CCK-8 assay at 0, 24, 48, 72, and 96 hours post-transfection with negative control siRNA (siNC) or three different siRNAs targeting HSP90AA1 (siHSP90AA1-1, -2, -3). **(C)** Representative images of colony formation assays for C918 and MUM-2B cells after HSP90AA1 knockdown. **(D)** Quantification of the number of colonies formed in each group, showing a significant reduction in the siHSP90AA1 groups. Data are presented as mean ± SD. *p < 0.05, **p < 0.01.

Functional assays revealed that HSP90AA1 silencing significantly inhibited cell proliferation. CCK-8 assays demonstrated a time-dependent decrease in viability post-transfection (**Figures 11A, B**). Colony formation assays further confirmed a significant reduction in both colony number and size (**Figures 11C, D**).

**Figure 12:**
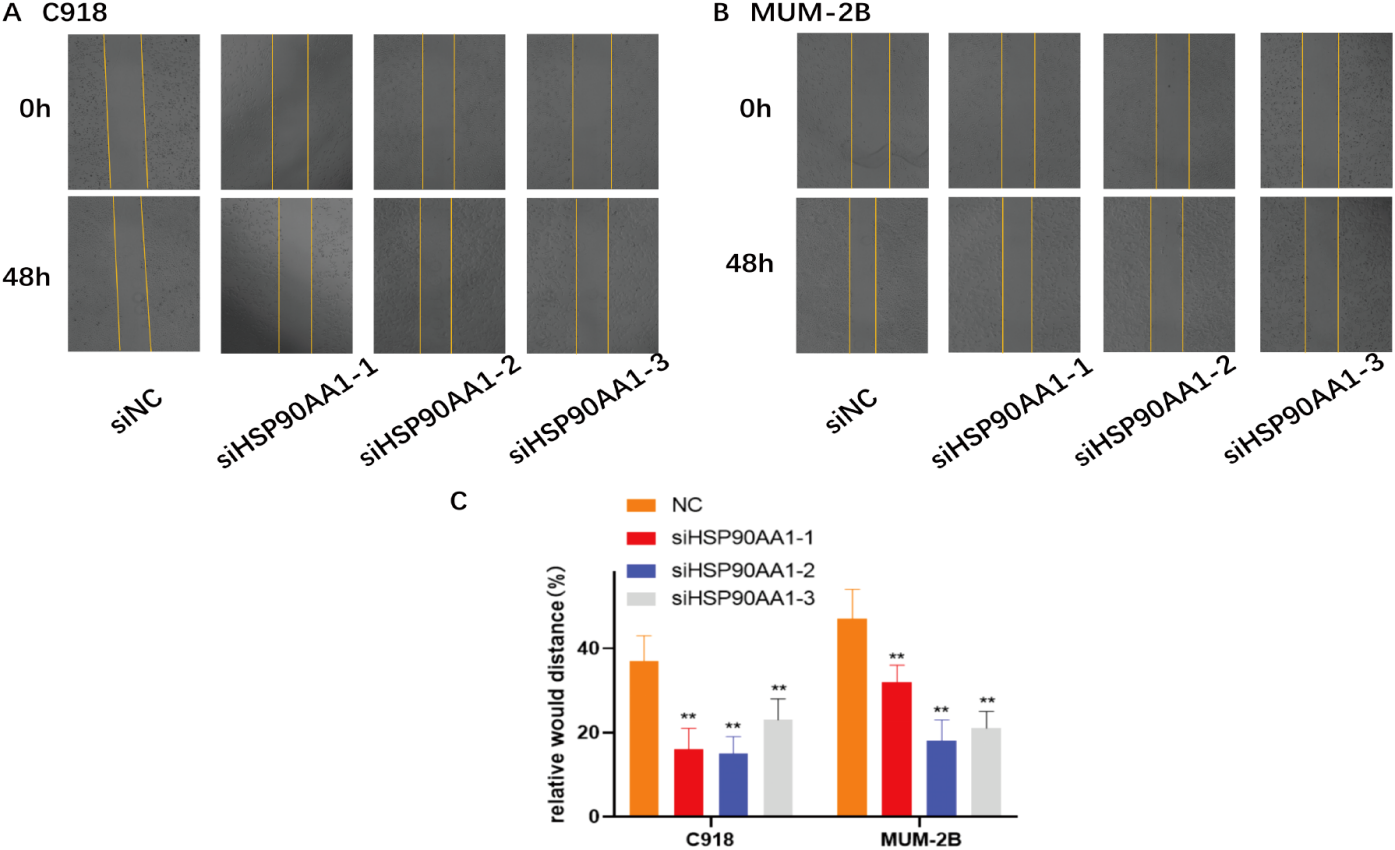
Knockdown of HSP90AA1 impairs UVM cell migration in a wound-healing assay. **(A, B)** Representative images of wound healing in C918 and MUM-2B cell monolayers at 0 and 48 hours after being scratched. Cells were transfected with either negative control siRNA (siNC) or siRNAs targeting HSP90AA1. **(C)** Quantitative analysis of the relative wound distance, showing significantly delayed wound closure in HSP90AA1-silenced cells compared to the control group. Data are shown as mean ± SD. **p < 0.01.

**Figure 13:**
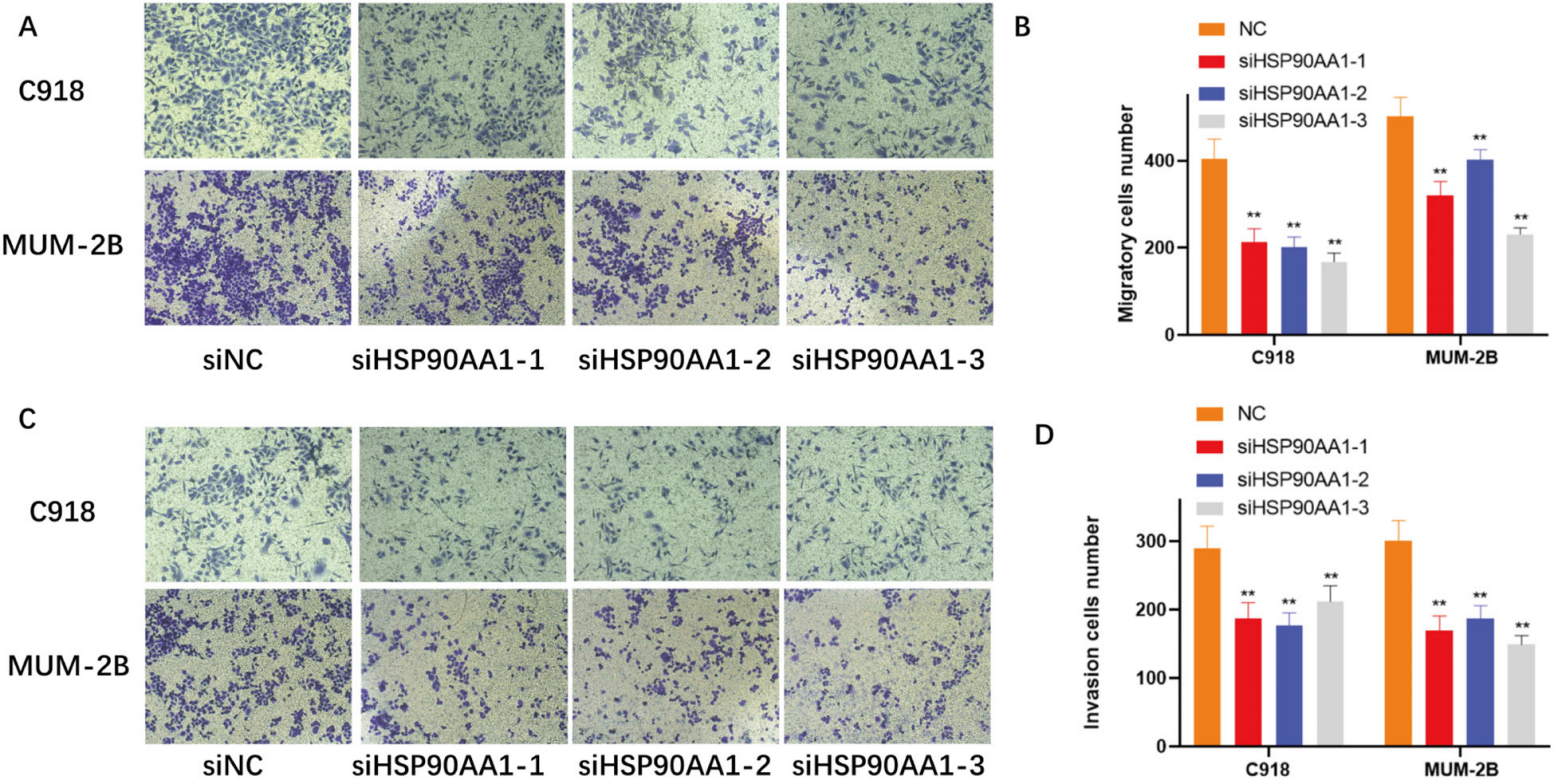
HSP90AA1 knockdown suppresses the migration and invasion of UVM cells. **(A)** Representative images of the Transwell migration assay for C918 and MUM-2B cells transfected with siNC or siHSP90AA1. **(B)** Quantification of the number of migratory cells per field. **(C)** Representative images of the Matrigel-coated Transwell invasion assay. **(D)** Quantification of the number of invasive cells per field. The results show that silencing HSP90AA1 significantly reduced both the migratory and invasive capacities of the cells. Data are shown as mean ± SD. **p < 0.01.

In addition, cell migration and invasion were markedly impaired. Wound healing assays showed delayed closure in knockdown cells (**Figures 12A–C**), and Transwell assays quantitatively verified decreased migration and invasion capacities (**Figures 13A–D**).

### 3.10 HSP90AA1 knockdown inhibits tumorigenicity *in vivo*

**Figure 14:**
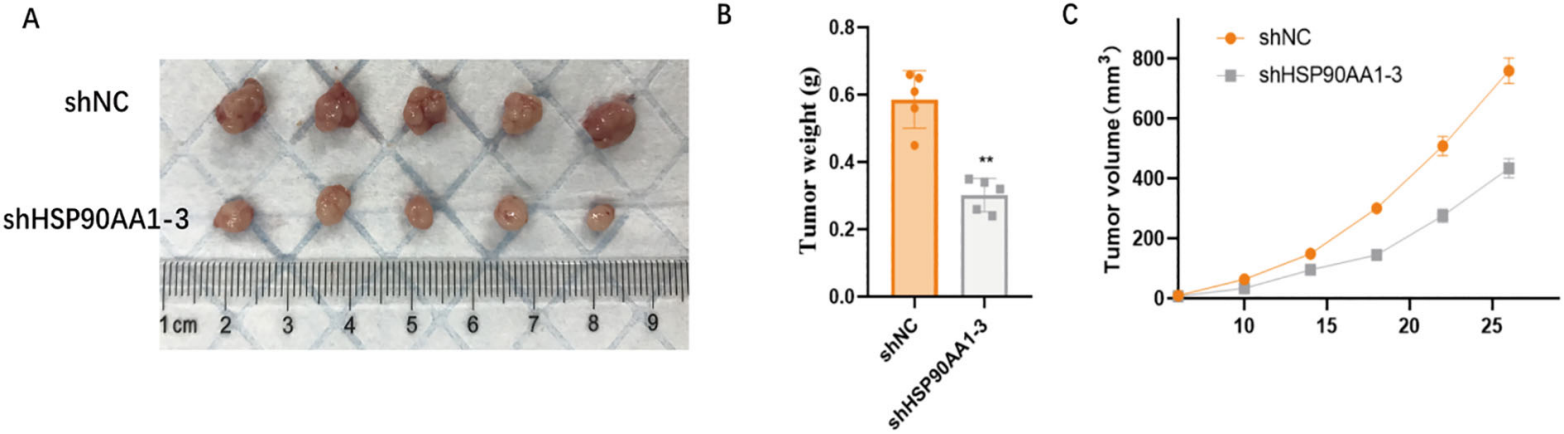
HSP90AA1 silencing inhibits UVM tumorigenicity in an *in vivo* xenograft model. **(A)** Images of tumors excised from nude mice 28 days after subcutaneous injection with C918 cells stably transfected with a negative control shRNA (shNC) or an shRNA targeting HSP90AA1 (shHSP90AA1-3). **(B)** Quantification of the final tumor weight, showing a significant reduction in the shHSP90AA1–3 group. **(C)** Tumor growth curves tracking tumor volume over the 28-day period, indicating slower growth in the HSP90AA1 knockdown group. Data are presented as mean ± SD. *p < 0.05, **p < 0.01.

To further confirm the oncogenic role of HSP90AA1 *in vivo*, a subcutaneous xenograft model was established in nude mice. C918 cells with stable HSP90AA1 knockdown (shHSP90AA1-3) exhibited significantly reduced tumorigenicity. Compared with the control (shNC) group, tumors in the knockdown group grew more slowly (**Figure 14C**). At the endpoint, both tumor weight and volume were markedly decreased (**Figures 14A–B**).

These *in vivo* findings corroborate the *in vitro* results, confirming that HSP90AA1 acts as a key driver of malignant progression in UVM.

### 3.11 HSP90AA1 knockdown modulates inflammatory and apoptotic pathways

**Figure 15:**
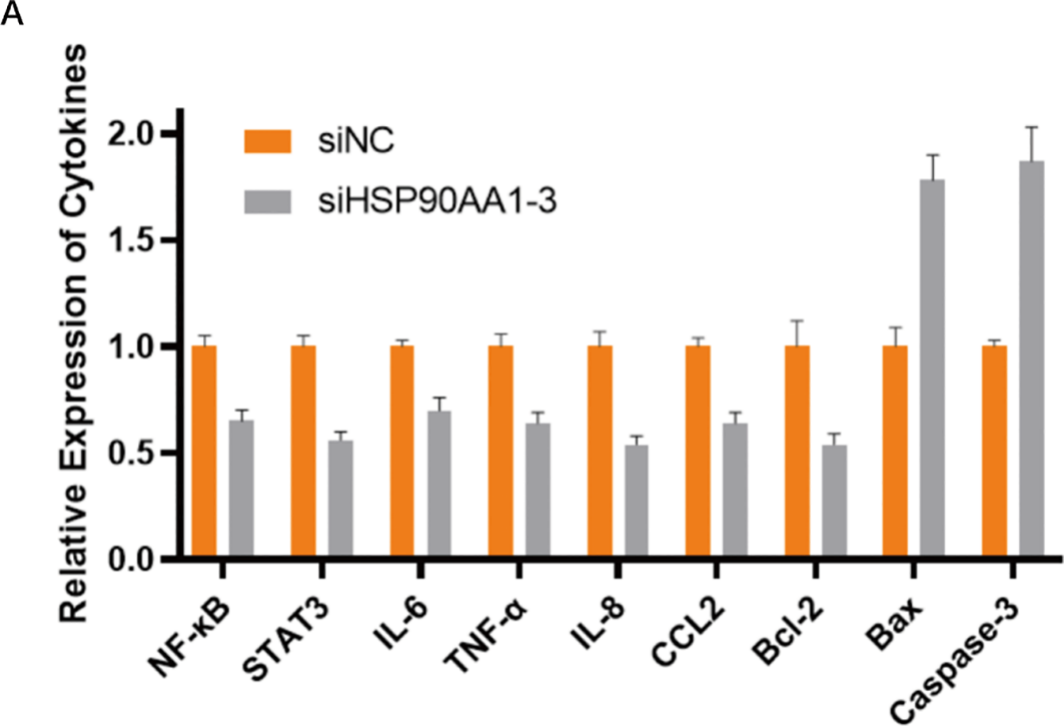
HSP90AA1 knockdown modulates inflammatory and apoptotic signaling pathways. **(A)** Bar chart showing the relative expression levels of key proteins involved in inflammation (NF-κB, STAT3, TNF-α, IL-6, IL-8, CCL2) and apoptosis (Bcl-2, Bax, Caspase-3) as measured by ELISA. Silencing of HSP90AA1 (siHSP90AA1-3) led to a decrease in pro-inflammatory and anti-apoptotic markers, and an increase in pro-apoptotic markers compared to the negative control (siNC).

To elucidate the molecular mechanisms underlying HSP90AA1-mediated oncogenic effects, changes in key pathway components were assessed by ELISA. HSP90AA1 silencing resulted in a marked reduction of pro-inflammatory cytokines and signaling molecules, including NF-κB, STAT3, TNF-α, IL-6, IL-8, and CCL2. Concurrently, the anti-apoptotic protein Bcl-2 was downregulated, while Bax and Caspase-3 levels were significantly increased (**Figure 15A**).

These results suggest that HSP90AA1 knockdown may exert its anti-tumor effects by suppressing inflammatory signaling and promoting apoptosis in UVM cells.

## 4 Discussion

In the clinical management of advanced cancers, including uveal melanoma (UVM), opioid analgesics such as hydromorphone serve as a double-edged sword: they are indispensable for pain control yet may biologically intersect with tumor progression pathways (16, 20–22). Rather than viewing this solely as a clinical paradox, we utilized this potential pharmacological overlap as a data-driven entry point to interrogate the UVM signaling landscape (23). By integrating computational network pharmacology with experimental validation, this study systematically screened for and identified HSP90AA1 as a core regulatory hub mediating inflammatory signaling and malignant progression in UVM.

Our investigation employed network pharmacology not merely to predict drug efficacy, but as a systems-level screening strategy to uncover hidden vulnerabilities within the UVM regulatory network. This approach successfully identified 44 potential targets shared between the opioid interaction network and UVM pathology. Subsequent topological analysis highlighted 10 hub genes occupying central positions, providing a prioritized list of candidates for biological validation. Functional enrichment analysis revealed that these hubs are heavily enriched in canonical oncogenic pathways, such as MAPK and PI3K–Akt signaling. This convergence is biologically significant because UVM is fundamentally driven by activating GNAQ and GNA11 mutations, which constitutively hijack these exact signaling cascades (11, 24–28). While the established UVM cell lines used in this study (C918 and MUM-2B) possess complex genetic backgrounds that may differ from primary tumors regarding specific *GNAQ/GNA11* mutational status, they nonetheless exhibit the high oncogenic stress and rapid proliferation characteristic of aggressive UVM. It is important to note that while HSP90AA1 is a common survival gene, UVM exhibits a unique ‘Chaperone Dependency’ distinct from other cancers. UVM is fundamentally driven by *GNAQ/GNA11* mutations, which constitutively hijack the MAPK and PI3K/Akt pathways. These hyperactivated kinases are well-known ‘client proteins’ that require HSP90 for folding and stability. Consequently, HSP90AA1 serves not merely as a generic hub, but as the essential infrastructure sustaining the specific oncogenic signaling driven by UVM-specific mutations. Targeting HSP90AA1 thus collapses the very signaling architecture that *GNAQ*-mutant cells rely on for survival.

Among the identified hubs, we prioritized HSP90AA1 for in-depth validation due to its unique role as a “master chaperone” in proteostasis. Unlike linear signaling kinases, HSP90AA1 functions as a structural infrastructure, stabilizing a vast array of oncogenic client proteins (e.g., EGFR, ERBB2, AKT) that overlap extensively with the hub genes identified in our network analysis (11, 17, 29, 30). This suggests that targeting HSP90AA1 could collapse multiple signaling axes simultaneously, offering a more robust therapeutic strategy than inhibiting single kinases (31–34).

A key finding of this study is the complex expression pattern of HSP90AA1 in UVM. We observed lower transcriptional levels of HSP90AA1 in tumor tissues compared to normal eye tissues. However, it is important to note that the “normal” control data from the GTEx database largely consists of retinal tissue, which is metabolically highly active, whereas UVM originates from choroidal melanocytes. This tissue-level metabolic discrepancy likely accounts for the apparent downregulation in tumors relative to normal controls. Therefore, the more clinically relevant finding is the intratumoral heterogeneity: within the UVM patient cohort, high HSP90AA1 expression strongly correlates with advanced disease and poor survival. We propose that this reflects an intrinsic “chaperone addiction” of aggressive UVM cells. Driven by chronic oncogenic signaling (e.g., MAPK hyperactivation from GNAQ mutations) and genomic instability, malignant cells experience elevated proteotoxic stress. Only tumor subclones capable of upregulating HSP90AA1 can maintain proteostasis and survive this stress, rendering high HSP90AA1 expression a hallmark of the most aggressive and resilient tumor populations (35–38).

Furthermore, our study elucidates the critical role of HSP90AA1 in shaping the Tumor Immune Microenvironment (TIME). We observed that high HSP90AA1 expression correlates with increased infiltration of CD8+ T cells and elevated pro-inflammatory chemokines (CXCL9, CXCL10). However, this “inflamed” phenotype paradoxically co-exists with poor prognosis, indicative of a state of immune dysfunction or exhaustion (39). Our experimental data provide a mechanistic explanation for this phenomenon. siRNA-mediated knockdown of HSP90AA1 in UVM cells significantly inhibited the NF-κB and STAT3 inflammatory pathways. Mechanistically, the physical interaction between HSP90 and its client proteins, including STAT3 and NF-κB, has been well-established in prior biochemical studies (40, 41). Consistent with this chaperone-client relationship, our results showed that silencing HSP90AA1 led to a marked reduction in the protein levels of NF-κB and STAT3. This degradation is a hallmark functional consequence of chaperone inhibition, confirming that HSP90AA1 is required to stabilize these key inflammatory transcription factors in UVM cells. This was evidenced by the reduced levels of key cytokines (TNF-α, IL-6, IL-8, CCL2) in the conditioned medium. We also noted a reduction in NF-κB and STAT3 levels in the supernatant; given that these are primarily intracellular transcription factors, their presence in the conditioned medium likely reflects leakage associated with cell turnover or death, serving as markers of the overall inflammatory burden of the tumor cell population. This blockade of inflammatory signaling was accompanied by suppressed proliferation, migration, invasion, and induced apoptosis *in vitro*, as well as attenuated tumorigenicity *in vivo*. Collectively, these results demonstrate that HSP90AA1 is not only a supporter of tumor cell survival but also a master regulator of the inflammatory milieu that may facilitate immune evasion.

Limitations: It is important to acknowledge the limitations of this study. First, the normal control data utilized from the GTEx database predominantly represents retinal tissue. Due to the inherent physiological and metabolic differences between the retina and the choroid (the origin of UVM), comparisons of absolute gene expression levels between “normal” and “tumor” samples should be interpreted with this context in mind. Second, while our *in vivo* xenograft model confirmed the role of HSP90AA1 in promoting intrinsic tumor growth, nude mice lack functional T cells. This model was chosen to isolate tumor-intrinsic proliferation from adaptive immune responses. However, to bridge this gap, we integrated analysis of human scRNA-seq data, which confirmed the presence of recruited T cells in the patient microenvironment, with *in vitro* assays demonstrating the HSP90AA1-dependent secretion of recruitment chemokines (e.g., CCL2, IL-8). Thus, our findings suggest that HSP90AA1 creates the *potential* for immune modulation, a hypothesis supported by clinical transcriptomic patterns. Therefore, the immunomodulatory effects of HSP90AA1 discussed herein are primarily inferred from our single-cell analysis and *in vitro* cytokine assays, and warrant further verification in immunocompetent models. While our network pharmacology analysis identified HSP90AA1 based on its connectivity with hydromorphone targets, our experimental validation focused strictly on the intrinsic function of HSP90AA1 in UVM cells using knockdown models. We did not directly evaluate the pharmacological effects of hydromorphone on HSP90AA1 expression or activity in this study. Therefore, whether clinical opioid usage directly exacerbates this “chaperone addiction” *in vivo* remains a hypothesis generated by our computational model that requires future pharmacological verification. Nevertheless, our findings definitively establish HSP90AA1 as a critical, independent therapeutic target for UVM, regardless of the upstream trigger.

In conclusion, by pivoting from computational screening to biological verification, we successfully decoded HSP90AA1 as a pivotal driver of the UVM inflammatory and immune microenvironment. Our findings support a model of “Intrinsic Chaperone Dependency,” where aggressive UVM cells rely on HSP90AA1 to buffer oncogenic stress and sustain inflammatory signaling. Targeting HSP90AA1 represents a promising strategy to dismantle this dependency and remodel the immunosuppressive microenvironment in UVM.

## 5 Conclusion

This study successfully employed a network-based screening strategy combined with experimental validation to decode the key regulators of the uveal melanoma (UVM) microenvironment. Our computational analysis identified a core gene signature associated with inflammatory signaling and pinpointed the molecular chaperone HSP90AA1 as a central regulatory node. We elucidated that high intratumoral HSP90AA1 expression significantly correlates with advanced disease and poor survival, a phenomenon we attribute to “chaperone addiction” in aggressively proliferating tumor cells striving to maintain proteostasis. Our *in vivo* xenograft experiments confirmed that silencing HSP90AA1 significantly suppresses tumorigenicity. Furthermore, *in vitro* assays validated the underlying mechanism, showing that HSP90AA1 knockdown inhibits proliferation and invasion by suppressing NF-κB and STAT3 inflammatory signaling and promoting apoptosis. These findings support a conceptual model of “Intrinsic Chaperone Dependency,” where UVM cells rely on HSP90AA1 to buffer the proteotoxic stress generated by chronic oncogenic signaling. Consequently, this work identifies HSP90AA1 as a critical, independent therapeutic target. These results suggest that targeting HSP90AA1 offers a promising strategy for potentially remodeling the tumor immune microenvironment and overcoming therapeutic resistance in aggressive UVM.

## Data Availability

The datasets presented in this study can be found in online repositories. The names of the repository/repositories and accession number(s) can be found in the article.
